# Over-Generalizing About GC (Hypoxia): Pitfalls of Limiting Breadth of Experimental Systems and Analyses in Framing Informatics Conclusions

**DOI:** 10.3389/fimmu.2021.664249

**Published:** 2021-05-10

**Authors:** Mark R. Boothby, Ariel Raybuck, Sung Hoon Cho, Kristy R. Stengel, Volker H. Haase, Scott Hiebert, Jingxin Li

**Affiliations:** ^1^ Department of Pathology, Microbiology & Immunology, Molecular Pathogenesis Division, Vanderbilt University Medical Center and School of Medicine, Nashville, TN, United States; ^2^ Department of Biochemistry, Vanderbilt University School of Medicine, Nashville TN, United States; ^3^ Department of Medicine, Nephrology Division, Vanderbilt University Medical Center and School of Medicine, Nashville, TN, United States; ^4^ Medical Scientist Training Program, Perelman School of Medicine, University of Pennsylvania, Philadelphia, PA, United States

**Keywords:** hypoxia, intermediary metabolism, Germinal center (GC) B cells, RNA-Seq, polyclonal preimmune repertoire, BCR transgenic mice

## Abstract

Accumulating evidence suggests that many immune responses are influenced by local nutrient concentrations in addition to the programming of intermediary metabolism within immune cells. Humoral immunity and germinal centers (GC) are settings in which these factors are under active investigation. Hypoxia is an example of how a particular nutrient is distributed in lymphoid follicles during an antibody response, and how oxygen sensors may impact the qualities of antibody output after immunization. Using exclusively a bio-informatic analysis of mRNA levels in GC and other B cells, recent work challenged the concept that there is any hypoxia or that it has any influence. To explore this proposition, we performed new analyses of published genomics data, explored potential sources of disparity, and elucidated aspects of the apparently conflicting conclusions. Specifically, replicability and variance among data sets derived from different naïve as well as GC B cells were considered. The results highlight broader issues that merit consideration, especially at a time of heightened focus on scientific reports in the realm of immunity and antibody responses. Based on these analyses, a standard is proposed under which the relationship of new data sets should be compared to prior “fingerprints” of cell types and reported transparently to referees and readers. In light of independent evidence of diversity within and among GC elicited by protein immunization, avoidance of overly broad conclusions about germinal centers in general when experimental systems are subject to substantial constraints imposed by technical features also is warranted.

## Introduction

In the March 2020 issue of *Nature Immunology*, Weisel, Shlomchik, and co-workers presented interesting data pioneering the use of flow-purified B cells from BCR knock-in mice to explore substrate utilization and metabolic features of B lymphocytes (1). These included naïve B cells - in some but not all comparisons - and a population of germinal center (GC)-phenotype B cells recovered from recipients with a monomorphic B cell population designed to avoid inclusion of other B cells into GC (1–3). Comparisons also involved B cells after T-independent activation *in vivo* (1). In light of the limits to using bio-informatic data to reach conclusions about biological systems, the new evidence about fatty acid oxidation (1) advances insights beyond gene expression profiles comparing naïve and GC B cells (4). However, the paper evoked a need to evaluate the conclusive statement that the “GCBC transcriptome is not commensurate with [….] hypoxia” and similar broad conclusions of the text. This claim seems connected to a view of the authors that RNA-Seq data with GCBC do not contain evidence of enrichment for genes encoding glycolytic enzymes, or that such increases relative to naïve B cells would necessarily show up in a metabolomics analysis with ^13^C-labeled glucose. These issues prompted examination of these and other data sets in GEO. The results of the analyses point to limits to the conclusions as stated in (1); they also raise a broader question about the system used for this work.

Several papers (4–6) have documented results from intravital labeling with imidazole compounds that covalently modify cellular constituents when the mitochondria of viable cells operate under reductive conditions due to intracellular hypoxia (7–9). Work under controlled conditions has shown that a meaningful signal above background is obtained only when the ambient pO_2_ is below about 1-1.5%, levels sufficient to yield HIF stabilization (7–9). Indeed, direct evidence of increased HIF-1α has been presented for both GC B cells (4, 5) and their Tfh counterparts (10), suggesting that many GC light zones are hypoxic. Of note, any issue of HIF function requires understanding that BCR engagement and TLR stimulation cause sustained HIF-1α and HIF-2α stabilization, which presents a drawback to comparing activated versus GC B cells. It might formally be possible that duration of the hypoxia, BCR signaling, and HIF stabilization failed to yield changes in mRNA concentrations large enough for enough gene products to yield a “statistically significant” result in a gene set enrichment analysis (GSEA) algorithm. Indeed, using a gene signature derived with the human breast cancer-like cell line MCF7 (11), the authors’ analysis of their purified GC B cells suggested that neither hypoxia-related nor HIF-1 target genes were enriched under their conditions of experimentation (1). Our previous published work had used GSEA with a gene signature indicative of biologically significant hypoxia (12) and scored the result of 2-fold normalized enrichment as statistically significant (4). In contrast, application of this gene signature to the data sets of (1) yielded a balanced mix of increased versus decreased mRNA and was not “statistically significant”. This difference along with other disparities of the data prompted us to compare the informatic findings while also using additional benchmarks that could test each report for independent replication.

## Methods and technical log

Datasets comparing RNA expression data from “naïve” (IgD+ or Follicular) versus immunization-induced GC B cells were mined from the GEO depositions of raw sequencing data, all of which were generated with Illumina Hi-Seq 2000 or 2500 instruments. For a bespoke pipeline, sequences were trimmed using the fastp FASTQ preprocessor for overrepresented sequences and to remove any sequence with a quality score <10 (13). Trimmed sequences were aligned to the mm10 mouse genome using the STAR sequence aligner of Dobin et al. (14). Aligned sequences were then quality-tested using Qualimap (15) software, and counted using featurecounts in Rsubread (16). To cross-check results and to use a commercial platform readily accessible to anyone seeking to re-analyze the data herein, the RNA-Seq platform within the suites of Basepair Technologies were used. Processing of the gene expression and PCA were cross-checked by using the commercial pipeline of Basepair Technologies as applied to the primary data for naïve and GC B cells of the papers cited as (1, 4, 17, 18).

For heatmap generation (**Figures 2A, B**) *via* the Basepair Technology platform, first the raw read counts generated using STAR aligner and featurecounts were normalized using the DESeq2 package. DESeq2 (19) performs an internal normalization where geometric mean is calculated for each gene across all samples. The counts for a gene in each sample are then divided by this mean. The median of these ratios in a sample is the size factor for that sample. After this, a Z-score normalization is performed on the normalized read counts across samples for each gene, so that Z-scores are computed on a gene-by-gene (row-by-row) basis by subtracting the mean and then dividing by the standard deviation. Computed Z scores were then used to plot heatmaps in which each row represents one gene. There were no substantive differences between different data sets in the quality [for naïve and GC B cell RNA respectively, 92% and 91-92%, 95% and 94-95%, 94% and 93%, and 87% and 91% for the papers referenced in the main text as (1, 4, 13, and 14, respectively) *via* the in-house pipeline uniformly applied to all samples. Comparable values, all above 90%, were generated by the Basepair Technologies pipeline and STAR alignment algorithms. Length scoring also was indistinguishable among the different datasets, two of which [the papers cited as (1) and (18) in the main text] were generated by paired-end sequencing. These steps were followed by multi-factor differential expression analysis in which deposited datasets as well as “naïve” *vs.* “GCB” expression data were compared using DESeq2 (20). Effect size shrinkage of differential expression data was normalized using the *apeglm* method published by Zhu et al. (21). Euclidean distances and Spearman correlation coefficients were derived by applying a standard “dist” function in R to the data described above. Gene set enrichment analyses were carried out using the GSEA program of the Broad Institute (22, 23) and gene sets derived from published literature, the Rat Genome database (“RGD”), and the Broad KEGG database. As a technical and data note, the informatics pipeline reported in (1) used voom instead of DESeq2, followed by rankSumTestWithCorrelation instead of GSEA. As expected from the technical literature (19, 24–27), the results from each pipeline did not materially differ when applied to the GEO-deposited data of (1). For **Figure 2D** display of count data in a manner that reduces dependence on the variance of the mean for low count data, blinded dispersion estimation using the variance stabilizing transform, VST (28), was conducted on the DESeq2 count data followed by generation of a heat map in which the count data are displayed rather than relative expression.

## Results

**Figure 1:**
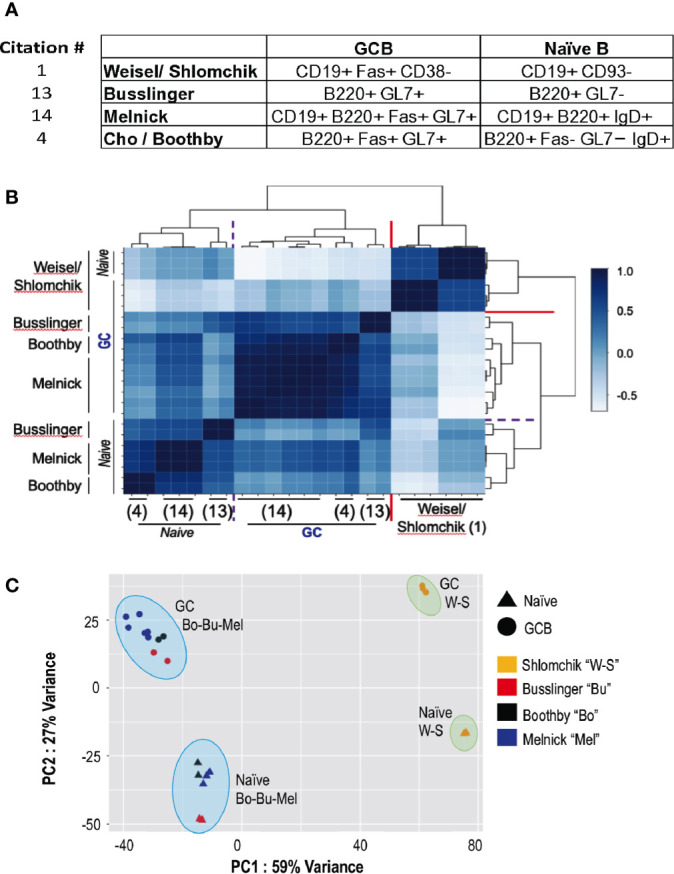
Quantitative comparisons of the overall RNA-Seq data for naïve and GC B cells in the transgenic and non-transgenic systems. Raw RNA-Seq data for naïve and GC B cells were downloaded from the GEO deposits for the papers cited as (1), “W-S” (17);, “Bu” (4);, “Bo” (18); “Mel”, and put through each of two separate analysis pipelines applied uniformly to all data (detailed in the Methods and technical log). **(A)** A tabulation of the surface markers used to purify naïve (or naïve follicular) and total germinal center B cells in the papers analyzed. **(B)** Self-organizing map from unsupervised clustering based on Spearman correlation coefficients across the data sets of the papers cited as (1, 4, 17, 18). Darker blue represents strong positive correlation (1.0 = identical across the RNA-Seq data); lightest blues are anti-correlated. **(C)** Shown here, a PCA plot depicting results across the datasets for the indicated conditions (triangles, naïve; circles, GC B cells) and datasets (color-coded as to the paper linked to each GEO data set according to the Legend) generated using the bespoke analysis pipeline detailed in the *Methods and Technical Log* (below). X- and Y- axes are defined by PC1 and PC2, accounting for 59% and 27% of the variance across the datasets, respectively. The results match those derived by the fully independent analysis using the default settings in Basepair Technologies’ pipeline (not shown).

GC hypoxia was observed with several types of immunization, including with NP-carrier (ovalbumin) (6), but only one paper (4), by Cho, Boothby, et al., had RNA-Seq data for comparisons. However, contemporaneous (2016, 2017) papers with data deposited in GEO had replicate data on naïve and bulk GC B cells (i.e., unfractionated mixtures of all of the diverse types of GC B cell, **Figure 1A**) in a similar time frame (7-10 d) after immunization with the same immunogen (SRBC) (17, 18). We analyzed the sequencer output data for all four papers (1, 4, 17, 18) using the same two parallel pipelines (one assembled in-house, detailed in a technical log appended to this Perspective; a second *via* Basepair Technologies, Inc for an independent framework).

**Figure 2:**
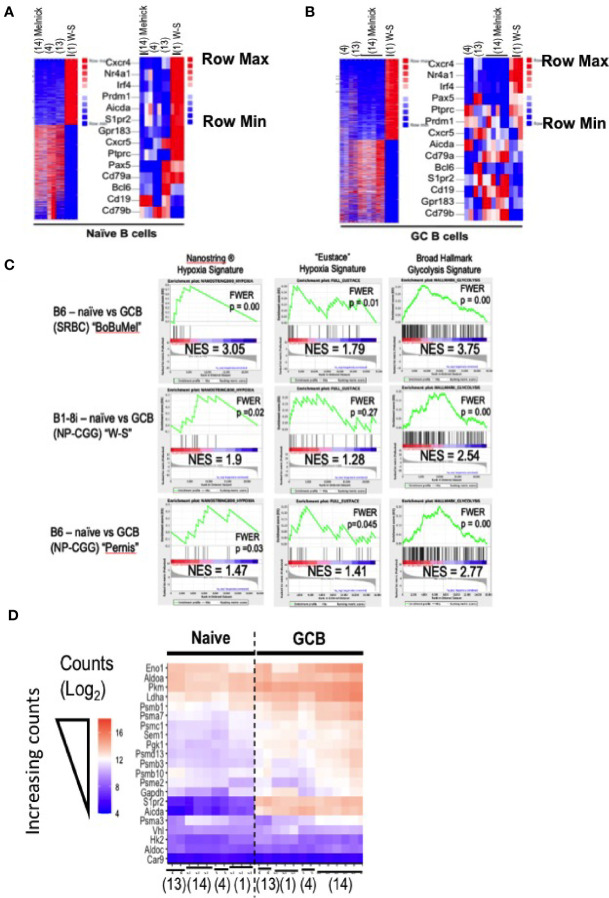
Comparisons of condition-dependent differential gene expression. As in **Figure 1**, GEO data deposits were downloaded for the papers cited as (1), “W-S” (17);, “Bu” (4);, “Bo” (18); “Mel” and processed by trimming, alignment, generation of normalized counts (FPKM), and quantification of differential expression using each of two independent pipelines (i.e., both bespoke and Basepair Technologies’). **(A, B)** Heat maps generated in the Basepair Technologies platform and derived from differential expression analyses comparing the groups “SRBC-immunized B6 mice” versus the BALB/c B1-8i, Vk -/- system are shown for **(A)** naïve and **(B)** GC B cells. In each panel, all differentially expressed genes (16% and 14% of totals, respectively, i.e., 3214/22,843 and 3461/21,327 genes differentially expressed >2-fold with p-adjusted < 0.05) are displayed. To the right, a subset of B lineage-specific or other genes functionally relevant in GC biology are shown. In these heat maps, entries that are dark red are upregulated and those that are blue are downregulated. Since the rows (genes) are Z-Score scaled, the maps report differences in expression of single genes across the samples. **(C)** Gene Set Enrichment Analyses (GSEA) were performed using the Broad algorithm as detailed in the Methods log. Analyses were performed both with data derived by the bespoke pipeline (shown here) and with the expression data exported from the Basepair pipeline, which yielded congruent results to those shown here. Inset values show the normalized enrichment score (NES) and p value after compensation for multiple comparisons (FWER), as indicated. Each panel displays results for a separate analysis in which ranked differential expression data were processed using the Broad Institute’s GSEA software. The three columns represent outputs obtained using the indicated gene signatures: Hypoxia Signature as established by Nanostring Technologies ^®^, the hypoxia gene signature of Eustace et al. [as in references (4, 12)], and the Broad Institute’s Hallmark Glycolysis gene set. The rows of panels identify the RNA-Seq data sets that were analyzed in comparisons of GC to naïve B cells: (i) data from like samples in (4, 17, 18); (ii) data from (1); and (iii) data from (29), all of which were processed through the same pipeline with same parameters.** (D)** A heatmap of the blinded variance-stabilizing transformed (VST) count data for selected genes encoding glycolytic enzymes or hypoxia-related genes in all samples [naïve and GC B cells of (1, 4, 17, 18)], benchmarked against two genes (*Aicda*; *S1pr2*) known to be highly expressed in GC B cells. Color coding from lowest (darker purple) counts to highest (deepest peach) is as indicated. Columns position (placement and order) was the computational result of self-organizing mapping, with expected separationi of naïve from GC B cells. Citations below the heat map indicate the publication sourcing of each column’s data.

Several salient observations emerged from these comparisons. Unsupervised clustering with Spearman correlation analyses (**Figure 1B**), Principal Components analyses (**Figure 1C**), and the Euclidean distances among the different types of samples, revealed that the results from (1) differ substantially from the data of (3), (17), and (18). *First*, the mRNA expression pattern of GC B cells generated after transferring large numbers of B cells [apparently, 10^6^ - (2) as cited in (1)] biased toward a single specificity BCR (B1-8^i^ Vκ -/-) into recipient mice with a monomorphic B cell population specific for Ig (AM14-Tg × V_κ_8R-KI BALB/c mice, i.e., specific for allotype-disparate IgG2a^b^) differed substantially from the other samples. A measure of overall differences between naïve and GC B cells was less in (1) (mean Euclidean distances of 126.3 ± 0.69) than the difference between the GC B cells of the transferred B1-8i, Vκ-/- cells (1) and the polyclonal GCBC (4, 17, 18) (mean Euclidean distances of 170.3 ± 10.3; p<0.01) as well as those of non-transgenic naïve versus GC B cells (147.6 ± SEM of 3.3; p<0.05). *Second*, the GC B cells from mice with a normal pre-immune repertoire (4, 17, 18) were substantially more similar to one another in comparing among three independent analyses (correlation coefficients in the range of +0.41 to +0.92) as opposed to anti-correlation of the B1-8i-derived GCBC, (correlation coefficients of -0.05 to -0.32). Although each of the independent data sets with the non-transgenic C57Bl/6 (B6) system differed somewhat – internally and from each other - the RNA-Seq “fingerprints” of naïve B cells in the polyclonal system were quite similar. Thus, the data were replicable when comparing independent SRBC immunizations of mice with no restrictions of the repertoire or BCR. In contrast, even the naïve B1-8^i^ Vκ-/- B cells were quite distinct from the clusters of polyclonal naïve B cells (**Figures 1B, C**). Relative to the data from (18) as well as (4), the changes were more modest in (1). The similarities and differences among independent data sets can also be parsed by heat maps of differentially expressed genes among the data sets, analyzing either the total set thereof or highlighting specific genes that are known to encode major determinants of B cell positioning, signal initiation during activation, of differentiation into GC B or plasma cells (**Figures 2A, B**). In practice, the collective data (**Figures 1B, C** **;** **2B**) indicate that the GCBC generated after adoptive transfers of B cells with a restricted repertoire into mice whose endogenous B cells will contribute little to the GC are qualitatively distinct from a polyclonal response that evolved from a polyclonal repertoire.

Turning to the question, are there hypoxia-related gene signatures in GC B cells when taking into account other work with a polyclonal repertoire, GSEA algorithms were applied using several different gene sets for hypoxia (**Figure 2C**). A further facet of what the analyses revealed is that with some hypoxia modules, even the RNA-Seq data of (1) show significant enrichment in GSEA (**Figure 2C**). These increases can also be appreciated in data with the actual counts when displaying all naïve and GC B cell data (**Figure 2D**). Moreover, RNA-Seq data with GC from NP-CGG-immunized mice with a normal pre-immune landscape (29) also show enrichment for a functional hypoxia signature (**Figure 2C**, data from the Pernis lab), and divergence from the B1-8i data. Metabolism rather than hypoxia was the central point of (1), a part of which was indirect evidence about glycolytic rates - perhaps on the view that if HIF were stabilized, expression of glycolytic genes should be increased. Prior work had provided evidence that the “glycolysis”, “mitochondrial respiration/oxidation” and “FAO” transcriptomes were increased in GCBC when compared to naïve B cells (4). Although this analysis was not evident in the Figure presented in (1) using the B1-8i system, a GSEA comparing naïve and GCBC with the RNA-Seq data in (1) found significant increases in glycolysis-related gene expression (**Figure 2C**), which also applied to each of the other data sets. Indeed, expression of genes encoding enzymes along the glycolysis pathway increased in GC B cell data sets of (1) as well as the polyclonal B6 GC B cells when compared to naïve B cells (**Figure 2D**).

## Discussion

The data presented here underscore that what is measured for an overall population of GC B cells is influenced by the structure of the experiment and the inputs analyzed. Key findings are that the cells analyzed in (1) were distinct from and changed less from naïve to GC phenotype than what is found in reproducible data with B cells that derived from a normal polyclonal repertoire (1, 17, 18). B1-8i, Vκ-/- B cells started out with substantial differences in activation markers such as *Nr4a1* and differentiation-related genes, i.e., *Prdm1* and *Irf4*. Of note, the differences do not relate to the particular informatic tools but instead to the selection of inputs. Thus, the pipelines used here yield similar results to those of (1) if, and only if, the analyses are restricted to those data sets and only use the selections in that work. Several factors may be involved in the differences between GC B cells in (1) from those in analyses published several years previously (4, 17, 18). Akin to findings with T cells (30), the experiments in (1) may involve the intra-clonal competition shown to result from using hundreds of thousands of transferred antigen receptor-transgenic cells (31, 32) - as already documented for B1-8i (31). In addition, the metabolic environments of highly diverse GC may differ from the secondary follicles that ensued with the transfer system used in (1). These factors warrant investigation, but the differences emphasize why it is vital that stated conclusions be restricted by limitations of the approach – not least when the approach is quite artificial.

GC are quite heterogeneous (33, 34). This point suggests that caution is warranted from the outset, militating against framing conclusions as blanket generalizations to apply across all GC. In terms of hypoxia or HIF gene signatures, prior work reported (4) that as many as 20% of splenic GC had no signal of intravital hypoxia, a variegation observed in parallel by others (J. Jellusova, personal communication). An integrative possibility is that hypoxia and/or its influence are reduced in GC designed to minimize bystander B cell involvement and dominated by a single specificity, as in (1–3). One technical issue is that there is no validated universal ‘hypoxia’ or ‘HIF’ module for activated lymphocytes, let alone a unitary module that includes GC B cells. Hypoxia and HIF responses are protean, but three decades of papers address cell type-specific functionality of a transcription factor. Thus, some restraint may be warranted before concluding that a GC transcriptome is not commensurate with hypoxia or conflating HIF with a particular magnitude of increased expression of genes encoding the enzymes of glycolysis or glycolytic flux. The issues connected to these questions take on added currency in considering antibody diversification during the persistent hypoxemia of many patients with severe CoVID-2019 infection. Thus, a great challenge will be to assess if human GC (in which questions about hypoxia, HIF, and *in situ* metabolism) exhibit effects such as those uncovered in mice (4–6) - either in normoxemic people or during concurrent hypoxemia.

Of course, such studies may rely heavily on scRNA-Seq and informatics analyses – conflicting or not – cannot settle secondary issues such as glucose uptake and utilization. Translation efficiency, post-translational modifications, and complex but unknown aspects of nutrient supplies, substrate concentrations, and substrate competition along webs of interconnected pathways all are downstream from the mRNA in question. That simply means that direct rigorous biochemical assays of glucose uptake per cell [e.g., ^3^H-2-deoxyglucose (DG), since 2-NBDG is known not necessarily to correlate with glucose entry rates (35)] and of glucose oxidation are needed before stating or accepting broad conclusions about germinal center B cells based on a single or special case as in (1). In this respect, normalizing a measure such as uptake to cell size – which is distinct from suitable compensation for autofluorescence in flow cytometry - lacks rigor. Normalization to size is analogous to concluding that a 160 kg person eating 4400 kCal/d has the same energy intake as one who is 80 kg and eats 2200 kCal/d, or claiming that a truck does not consume more fuel than a compact sedan if the size-normalized fuel consumption is the same. With respect to conclusions based solely on informatics, it is proposed that a reasonable standard is to (a) compare new RNA-Seq data sets to independent entries in GEO, and (b) quantify the correlation to - or difference from - those that are in GEO and readily comparable (i.e., not micro-array to RNA-Seq). Of essential importance, some restraint in statement of conclusions, along with openness about limitations, is vital at a time when the societal landscape is roiled by consequences of how scientists frame their work or evidence.

## Data Availability Statement

The datasets presented in this study can be found in online repositories. The names of the repository/repositories and accession number(s) can be found in the article/**Supplementary Material**.

## Author Contributions

MB wrote the text and guided analyses along with getting presubmission input from three independent faculty/PIs. AR led the primary informatic analyses, prepared figures, drafted portions of text, and edited the overall text. SC, KS, and JL performed analyses and provided insights into the RNA-Seq data and gene sets in the papers meta-analyzed. VHH provided input and guidance to SHC and MB; SHC provided input and guidance to KS and MB. All authors contributed to the article and approved the submitted version.

## Funding

Departmental Funds - Department of Pathology, Microbiology, & Immunology, Vanderbilt University Medical Center.

## Conflict of Interest

The authors declare that the research was conducted in the absence of any commercial or financial relationships that could be construed as a potential conflict of interest.

## References

[1] Weisel FJ Mullett SJ Elsner RA Menk AV Trivedi N Luo W. Germinal Center B Cells Selectively Oxidize Fatty Acids for Energy While Conducting Minimal Glycolysis. Nat Immunol (2020) 21:331–42. 10.1038/s41590-020-0598-4 PMC7112716 32066950

[2] Zuccarino-Catania GV Sadanand S Weisel FJ Tomayko MM Meng H Kleinstein SH. CD80 and PD-L2 Define Functionally Distinct Memory B Cell Subsets That are Independent of Antibody Isotype. Nat Immunol (2014) 15:631–7. 10.1038/ni.2914 PMC4105703 24880458

[3] Weisel FJ Zuccarino-Catania GV Chikina M Shlomchik MJ. A Temporal Switch in the Germinal Center Determines Differential Output of Memory B and Plasma Cells. Immunity (2016) 44:116–30. 10.1016/j.immuni.2015.12.004 PMC4724390 26795247

[4] Cho SH Raybuck AL Stengel K Wei M Beck TC Volanakis E. Germinal Centre Hypoxia and Regulation of Antibody Qualities by a Hypoxia Response System. Nature (2016) 537:234–8. 10.1038/nature19334 PMC5161594 27501247

[5] Jellusova J Cato MH Apgar JR Ramezani-Rad P Leung CR Chen C. Gsk3 is a Metabolic Checkpoint Regulator in B Cells. Nat Immunol (2017) 18:303–12. 10.1038/ni.3664 PMC5310963 28114292

[6] Abbott RK Thayer M Labuda J Silva M Philbrook P Cain DW. Germinal Center Hypoxia Potentiates Immunoglobulin Class Switch Recombination. J Immunol (2016) 197:4014–20. 10.4049/jimmunol.1601401 PMC5123804 27798169

[7] Koch CJ Evans SM Lord EM. Oxygen Dependence of Cellular Uptake of EF5 [2-(2-Nitro-1H-Imidazol-1-Yl)-N-(2,2,3,3,3-pentafluoropropyl) Acetamide]: Analysis of Drug Adducts by Fluorescent Antibodies vs Bound Radioactivity. Br J Cancer (1995) 72:869–74. 10.1038/bjc.1995.426 PMC2034014 7547233

[8] Mahy P De Bast M Gallez B Gueulette J Koch CJ Scalliet P. In Vivo Colocalization of 2-Nitroimidazole EF5 Fluorescence Intensity and Electron Paramagnetic Resonance Oximetry in Mouse Tumors. Radiother Oncol (2003) 67:53–61. 10.1016/S0167-8140(03)00028-8 12758240

[9] Koch CJ. Importance of Antibody Concentration in the Assessment of Cellular Hypoxia by Flow Cytometry: EF5 and Pimonidazole. Radiat Res (2008) 169:677–88. 10.1667/RR1305.1 18494550

[10] Cho SH Raybuck AL Blagih J Kemboi E Haase VH Jones RG. Hypoxia-Inducible Factors (HIF) in CD4^+^ T Cells Promote Metabolism, Switch Cytokine Secretion, and T Cell Help in Humoral Immunity. PNAS U S A (2019) 116:8975–84. 10.1073/pnas.1811702116 PMC6500120 30988188

[11] Schödel J Oikonomopoulos S Ragoussis J Pugh CW Ratcliffe PJ Mole DR. High-Resolution Genome-Wide Mapping of HIF-binding Sites by Chip-Seq. Blood (2011) 117:e207–17. 10.1182/blood-2010-10-314427 PMC3374576 21447827

[12] Eustace A Mani N Span PN Irlam JJ Taylor J Betts GN. A 26-Gene Hypoxia Signature Predicts Benefit From Hypoxia-Modifying Therapy in Laryngeal Cancer But Not Bladder Cancer. Clin Cancer Res (2013) 19:4879–88. 10.1158/1078-0432.CCR-13-0542 PMC3797516 23820108

[13] Chen S Zhou Y Chen Y Gu J. Fastp: An Ultra-Fast All-in-One FASTQ Preprocessor. Bioinformatics (2018) 29(17):i884–90. 10.1093/bioinformatics/bty560 PMC6129281 30423086

[14] Dobin A Davis CA Schlesinger F Drenkow J Zaleski C Jha S. STAR: Ultrafast Universal RNA-seq Aligner. Bioinf (Oxford England) (2013) 29(1):15–21. 10.1093/bioinformatics/bts635 PMC3530905 23104886

[15] Okonechnikov K Conesa A Garcia-Alcalde F. Qualimap 2: Advanced Multi-Sample Quality Control for High-Throughput Sequencing Data. Bioinformatics (2015) 32(2):292–4. 10.1093/bioinformatics/btv566 PMC4708105 26428292

[16] Liao Y Smyth GK Shi W. The R Package *Rsubread* is Easier, Faster, Cheaper and Better for Alignment and Quantification of RNA Sequencing Reads. Nucleic Acids Res (2019) 47(8):e47. 10.1093/nar/gkz114 30783653 PMC6486549

[17] Wöhner M Tagoh H Bilic I Jaritz M Poliakova DK Fischer M. Molecular Functions of the Transcription Factors E2A and E2-2 in Controlling Germinal Center B Cell and Plasma Cell Development. J Exp Med (2016) 213:1201–21. 10.1084/jem.20152002 PMC4925024 27261530

[18] Béguelin W Rivas MA Calvo Fernández MT Teater M Purwada A Redmond D. EZH2 Enables Germinal Centre Formation Through Epigenetic Silencing of CDKN1A and an Rb-E2F1 Feedback Loop. Nat Commun (2017) 8(1):877. 10.1038/s41467-017-01029-x 29026085 PMC5638898

[19] Zhang ZH. A Comparative Study of Techniques for Differential Expression Analysis on RNA-Seq Data. PLoS One (2014) 9(8):e103207. 10.1371/journal.pone.0103207 25119138 PMC4132098

[20] Love MI Huber W Anders S. Moderated Estimation of Fold Change and Dispersion for RNA-seq Data With Deseq2. Genome Biol (2014) 15:550. 10.1186/s13059-014-0550-8 25516281 PMC4302049

[21] Zhu A Ibrahim JG Love MI. Heavy-Tailed Prior Distributions for Sequence Count Data: Removing the Noise and Preserving Large Differences. Bioinformatics (2018) 35(12):2084–92. 10.1093/bioinformatics/bty895 PMC6581436 30395178

[22] Subramanian A Tamayo P Mootha VK Mukherjee S Ebert BL Gillette MA. Gene Set Enrichment Analysis: A Knowledge-Based Approach for Interpreting Genome-Wide Expression Profiles. Proc Natl Acad Sci USA (2005) 102:15545–50. 10.1073/pnas.0506580102 PMC1239896 16199517

[23] Mootha VK Lindgren CM Ericksson KF Subramanian A Sihag S Lehar J. Pgc-1a-responsive Genes Involved in Oxidative Phosphorylation are Coordinately Downregulated in Human Diabetes. Nat Genet (2003) 34:267–73. 10.1038/ng1180 12808457

[24] Ackermann M Strimmer K. A General Modular Framework for Gene Set Enrichment Analysis. BMC Bioinf (2009) 10:47. 10.1186/1471-2105-10-47 PMC2661051 19192285

[25] Hung JH Yang TH Hu Z Weng Z DeLisi C. Gene Set Enrichment Analysis: Performance Evaluation and Usage Guidelines. Brief Bioinform (2012) 13(3):281–91. 10.1093/bib/bbr049 PMC3357488 21900207

[26] Schurch NJ Schofield P Gierlinski M Cole C Sherstnev A Singh V. How Many Biological Replicates are Needed in an RNA-seq Experiment and Which Differential Expression Tool Should You Use? RNA (2016) 22:839–51. 10.1261/rna.053959.115 PMC4878611 27022035

[27] Costa-Silva J Domingues D Lopes FM. Rna-Seq Differential Expression Analysis: An Extended Review and a Software Tool. PLoS One (2017) 12:12e0190152. 10.1371/journal.pone.0190152 PMC5739479 29267363

[28] Anders S Huber W. Differential Expression Analysis for Sequence Count Data. Genome Biol (2010) 11:R106. 10.1186/gb-2010-11-10-r106 20979621 PMC3218662

[29] Ricker E Chinenov Y Pannellini T Flores-Castro D Ye C Gupta S. Serine-Threonine Kinase ROCK2 Regulates Germinal Center B Cell Positioning and Cholesterol Biosynthesis. J Clin Invest (2020) 130:3654–70. 10.1172/JCI132414 PMC7324193 32229726

[30] Badovinac VP Haring JS Harty JT. Initial T Cell Receptor Transgenic Cell Precursor Frequency Dictates Critical Aspects of the CD8(+) T Cell Response to Infection. Immunity (2007) 26:827–41. 10.1016/j.immuni.2007.04.013 PMC1989155 17555991

[31] Le TV Kim TH Chaplin DD. Intraclonal Competition Inhibits the Formation of High-Affinity Antibody-Secreting Cells. J Immunol (2008) 181:6027–37. 10.4049/jimmunol.181.9.6027 PMC2922957 18941192

[32] Abbott RK Lee JH Menis S Skog P Rossi M Ota T. Precursor Frequency and Affinity Determine B Cell Competitive Fitness in Germinal Centers, Tested With Germline-Targeting HIV Vaccine Immunogens. Immunity (2018) 48:133–46. 10.1016/j.immuni.2017.11.023 PMC5773359 29287996

[33] Tas JM Mesin L Pasqual G Targ S Jacobsen JT Mano YM. Visualizing Antibody Affinity Maturation in Germinal Centers. Science (2016) 351:1048–54. 10.1126/science.aad3439 PMC4938154 26912368

[34] Mesin L Ersching J Victora GD. Germinal Center B Cell Dynamics. Immunity (2016) 45:471–82. 10.1016/j.immuni.2016.09.001 PMC5123673 27653600

[35] Sinclair LV Barthelemy C Cantrell DA. Single Cell Glucose Uptake Assays: A Cautionary Tale. Immunometabolism (2020) 2(4):e200029. 10.20900/immunometab20200029 32879737 PMC7116014

